# A New Targeted Lipidomics Approach Reveals Lipid Droplets in Liver, Muscle and Heart as a Repository for Diacylglycerol and Ceramide Species in Non-Alcoholic Fatty Liver

**DOI:** 10.3390/cells8030277

**Published:** 2019-03-22

**Authors:** Christina Preuss, Tomas Jelenik, Kálmán Bódis, Karsten Müssig, Volker Burkart, Julia Szendroedi, Michael Roden, Daniel F. Markgraf

**Affiliations:** 1 Institute for Clinical Diabetology, German Diabetes Center, c/o Auf’m Hennekamp 65, D-40225 Düsseldorf, Germany; Christina.Preuss@ddz.de (C.P.); Tomas.Jelenik@ddz.de (T.J.); Kalman.Bodis@ddz.de (K.B.); Karsten.Muessig@ddz.de (K.M.); Volker.Burkart@ddz.de (V.B.); Julia.Szendroedi@ddz.de (J.S.); Michael.Roden@ddz.de (M.R.); 2 German Center for Diabetes Research (DZD e.V.), D-85764 München, Neuherberg, Germany; 3 Division of Endocrinology and Diabetology, Medical Faculty, Heinrich-Heine University, D-40225 Düsseldorf, Germany

**Keywords:** non-alcoholic fatty liver, lipid-induced insulin resistance, diacylglycerol, ceramide, lipid droplet, type 2 diabetes

## Abstract

Obesity is frequently associated with excessive accumulation of lipids in ectopic tissue and presents a major risk factor for type 2 diabetes (T2D) and non-alcoholic fatty liver disease (NAFLD). Diacylglycerols (DAGs) and ceramides (CERs) were identified as key players in lipid-induced insulin resistance, typical for such diseases. Recent results suggest that the subcellular distribution of these lipids affects their lipotoxic properties. However, the subcellular dynamics of these lipids and the role of lipid droplets (LDs) as a potential storage site is not understood. Here, we developed a liquid chromatography triple quadrupole mass spectrometry (LC-MS/MS)-method for the rapid and simultaneous quantification of DAG and CER species in tissue sample fractions. The assay is characterized by excellent recovery of analytes, limit of quantification, accuracy and precision. We established a fractionation protocol that allows the separation of subcellular tissue fractions. This method was subsequently tested to measure the concentration of DAGs and CERs in subcellular fractions of human muscle and several mouse tissues. In a mouse model of NAFLD, application of this method revealed a prominent role for LDs as repository for lipotoxic DAG and CER species. In conclusion, the new method proved as a valuable tool to analyse the subcellular dynamics of lipotoxins, related to the pathogenesis of insulin resistance, T2D and NAFLD.

## 1. Introduction

Lipids present a versatile group of molecules with a broad spectrum of functions [1]. It is now well accepted that the dysregulation of lipid metabolism represents a hallmark of the most common metabolic diseases worldwide, such as obesity, type 2 diabetes (T2D) and non-alcoholic fatty liver disease (NAFLD) [2,3,4,5]. A common feature of these diseases is the excessive accumulation of lipids in ectopic tissues, that is, adipocytes, muscle and liver, ultimately causing insulin resistance at tissue- and whole body level [6,7]. At a cellular level, fatty acids are taken up and channelled into the triglyceride (TAG) synthesis pathway. The sequential reactions of this evolutionary conserved pathway are highly compartmentalized, with fatty acids (FAs) being activated and attached to glycerol-3-phosphate (G3P) in the endoplasmic reticulum (ER) [8,9]. Subsequent reactions in the ER lead to the formation of the lipid intermediate diacylglycerol (DAG) which can be further esterified to TAG. Continuous formation of TAG leads to the formation of ER-derived lipid droplets (LDs), specialized lipid storage organelles. Interestingly, DAG can be channelled into LDs for localized synthesis of TAGs on LDs [10,11]. Lipolysis of TAGs leads to the formation of DAGs which can be further hydrolysed to provide FAs for energy production or channelled back into the ER for membrane lipid synthesis or very-low-density lipoprotein (VLDL) synthesis in the liver [12,13]. Studies in the model organism yeast suggest that the channelling of DAG from LDs to the ER is a regulated, protein-mediated process [14]. Alternatively, FAs, such as palmitate, can be channelled into the ceramide (CER) synthesis pathway [15,16].

Despite the well-studied association between excessive lipid accumulation in muscle and liver and the pathogenesis of insulin resistance, the ultimate storage lipid TAG appears to be metabolically inert [17]. In contrast, several studies have implicated DAGs and CERs as mediators of lipid-induced insulin resistance. Excessive supply of tissues with FAs and subsequent uptake can lead to an increase in cellular CER levels. Accumulated CERs were shown to inhibit insulin signalling by reducing AKT activity via activation of protein phosphatase 2A (PP2A) or the atypical protein kinase C isoform zeta (PKCζ) [18,19,20]. In addition, circulating FAs bind to toll-like receptor 4 (TLR4), thereby triggering enhanced synthesis of CERs leading to insulin resistance via the activation of inflammatory pathways [21,22]. In muscle, DAGs were shown to accumulate upon increased FA flux and recruit novel PKC theta (nPKCθ) to the plasma membrane, leading to the inhibitory phosphorylation of IRS1, thereby inhibiting the insulin signalling cascade and ultimately glucose uptake [23,24,25]. The excessive flux of FAs towards liver is associated with hepatic accumulation of DAGs and activation of nPKCε, which affects insulin signalling by inhibiting the activation of IRS proteins [26]. Similarly, a recent study in a mouse model of NAFLD revealed an accumulation of DAGs and subsequent activation of nPKCε in heart [27]. Differences in the subcellular distribution of DAGs seem to affect its lipotoxic properties [24,28,29,30,31]. Unfortunately, most currently applied subcellular fractionation techniques are not sufficient to distinguish between cellular membranes but rather separate crude membrane mixtures from the cytosol. Accordingly, the accumulation of DAGs specifically to the plasma membrane, as suggested in current working models, has not yet been shown. Recent efforts lead to the development of more sophisticated fractionation protocols in a mouse model of NAFLD [32]. Whereas this study shed light on the subcellular dynamics at a protein level, data on the dynamics of lipotoxic and potentially diabetes-relevant lipids are still missing. In particular, the role of LDs in various tissues as potential storage site of lipotoxic lipid species and its contribution to lipid-induced, nPKC-mediated insulin resistance is not understood.

Here, we developed a targeted lipidomics approach to simultaneously analyse DAGs and CERs in subcellular fractions, that is, membrane, cytosol and LDs of tissue samples. Applying this approach to a mouse model of NAFLD, we show that LDs in multiple tissues represent a repository for lipotoxic DAG and CER species. This new method will serve as a tool to decipher i) the general role of LDs in metabolic disorders and ii) the cellular pathomechanisms underlying lipid-induced insulin resistance.

## 2. Material and Methods

### 2.1. Chemicals and Reagents

Methanol (hypergrade), chloroform (LC-MS grade) and water (LC-MS grade) were purchased from Merck (Darmstadt, Germany). 2-propanol and ammonium formate were purchased from Fisher scientific (Waltham, MA, USA). Diacylglycerol reference standards 1,3-di-9Z,12Z-octadecadienolyl)-glycerol, 1,2-dipalmitoyl-sn-glycerol, 1-palmitoyl-2- oleoyl-sn-glycerol, 1-stearoyl-2-arachidonyl-sn-glycerol, 1-stearoyl-2-linoleoyl-sn-glycerol, 1-octadecanoyl-2-hexadecanoyl-sn-glycerol; ceramide reference standards Cer d18:1/14:0, Cer d18:1/16:0, Cer d18:1/18:0, Cer d18:1/18:1 (9Z), Cer d18:1/20:0, Cer d18:2/24:0, Cer d18:1/24:1 (15Z) and internal standards (IS) 1,3(d5)-di-(diheptadecenoyl)-glycerol, Cer d18:1/17:0 were acquired from Avanti polar lipids (Alabaster, AL, USA). Diacylglycerol standards 1,3-dipalmitolein (rac), 1-Palmitin-3-Linolein (rac), 1-Stearin-3-Olein (rac) were purchased from LGC Standards (Wesel, Germany); 1,2-Dimyristoyl-sn-glycerol, 1,2-dioctadecanoyl-sn-glycerol from Cayman Chemical (Ann Arbor, MI, USA) and 1,3-di-(9E-octadecenoyl)-glycerol from Sigma Aldrich (Saint Louis, MO, USA). Protease inhibitor Cocktail (PIC) was purchased from Roche Diagnostics GmbH (Mannheim, Germany).

### 2.2. Animals

Transgenic mice, 22-weeks old, with NAFLD due to adipose tissue-specific overexpression of sterol regulatory-element binding protein-1c (SREBP-1c) and matched C57B16 controls were maintained under pathogen-free conditions on a 12-h light-dark cycle, received standardized diet (Ssniff M-Z Extrudat, 4.5% fat; SSNIFF Spezialdiäten GmbH, Soest, Germany) and water ad libitum. Mice were exsanguinated through cervical incision and sacrificed by cervical dislocation. Organs were snap frozen in liquid nitrogen for future analyses. All experiments were performed according to the guidelines for the care and use of animals (GV-SOLAS [Society for Laboratory Animal Science]) and approved by the local council of animal care in line with the requirements of the German Animal Protection Act.

### 2.3. Human Muscle Samples

Human skeletal muscle (m. vastus lateralis) sample was obtained by biopsy from a 37 year old, male participant with type 2 diabetes of the German Diabetes Study (GDS) at a five year follow-up examination. The study design and cohort profile of the GDS are described elsewhere [33]. All participants gave written informed consent before inclusion in the study (Clinical trial.gov registration no: NCT01055093), which was performed according to the Declaration of Helsinki and approved by the local ethics board of Heinrich Heine University, Düsseldorf, Germany.

### 2.4. Sample Preparation

Tissue samples were quick frozen in liquid nitrogen after sampling and stored at −80 °C. For extraction and purification of lipids 50 mg of muscle and heart tissue was homogenized in 500 µL of buffer A (20 mM mM Tris/HCL, 1 mM EDTA 0.25 mM EGTA, 250 mM Sucrose, pH 7.4 2 × PIC) using an IKA T10 basic Ultra Turrax (IKA; NC, USA) and a tight-fitting glass douncer (Wheaton, UK). Due to high levels of lipids in livers of NAFLD mice, 20 mg of liver tissue was homogenized in 10 mL of Buffer A and 0.5 mL was used for further steps. Internal standards were added and samples were transferred to centrifuge tubes, overlaid with 150 µL Buffer A, containing 3% sucrose and centrifuged for 1 h, 100,000× *g*, 4 °C. The floating LD layer was separated from the underlying cytosol fraction using a Centri-Tube slicer (Beckman Coulter, Brea, CA, USA). Lipids of LD, cytosol and membrane fraction were extracted according to Folch et al. [34], followed by solid phase extraction (Sep Pak Diol Cartridges; Waters, MA, USA). The resulting lipid phase was dried under a gentle flow of nitrogen and resuspended in 400 µL methanol. 

For analysis of the protein composition, the LD fraction was subjected to acetone precipitation and the resulting protein pellet was resuspended in buffer A. The protein concentration of all fractions was determined (Pierce Protein Assay kit, Thermo Fisher, Waltham, MA, USA). The indicated amount of protein was resuspended in sodium dodecyl sulfate (SDS) sample buffer, heated for 5 min at 95 °C and analysed by SDS-polyacrylamide gel electrophoresis (PAGE) and Western blotting. Anti-Calnexin (rabbit polyclonal, Enzo Life Sciences), anti-GAPDH (rabbit monoclonal clone D16H11, Cell Signalling) and anti-PLIN2 (mouse polyclonal B01P, Abnova) antibodies were used. 

### 2.5. Lipid Analysis by LC-MS/MS

The chromatographic separation of analytes was conducted using an Infinity 1290 Ultra-High Performance Liquid chromatography system (Agilent Technologies Inc., Santa Clara, CA, USA) and a reverse-phase Luna Omega C18 column, 50 × 2.1 mm, 1.6 µm (Phenomenex, Torrance, CA, USA) operated at 50 °C. The injection volume was 1 µL. A binary gradient was used consisting of 5 mM ammonium formate in water (Solvent A) and 5 mM ammonium formiate in methanol (solvent B) at the flow rate of 0.4 mL/min. The following gradient conditions were applied: 0 min 78%B, 1.5 min–2 min 78–93%B, 2 min–8 min 93–97%, 8 min–10 min 97–99%, 10 min–14 min 99%, 14 min–15 min 99–78%.

The analytes were measured as ammonium adducts (DAGs) or protonated adducts (CERs) using electrospray ionization (ESI) and detected by multiple reaction monitoring (MRM) on a triplequadrupole mass spectrometer (MS, Agilent 6495; Agilent Technologies, CA, USA) operated in positive ion mode. Mass transitions and MS parameters are shown in Table 1.

#### Calibration and Quantification of Lipid Species

Calibration was achieved by 8-point calibration curves, adding the indicated amounts (1–1000 ng DAG, 1–200 ng CER) of combined standard mixtures and IS (500 ng DAG, 100 ng CER) to calibration samples in buffer A. No differences between standard mixtures in buffer A and BSA-containing matrix samples were observed (data not shown). For quantification, the ratio between analyte and IS was used. Regression line was quadratic (weighed 1/x) for DAGs and linear (weighed 1/x) for CER. Data analysis was performed using Masshunter Workstation software (Agilent Technologies Inc., Santa Clara, CA, USA). Data were exported into Excel and further processed.

### 2.6. Method Validation

#### Recovery

For the determination of extraction efficiency, we prepared homogenate from human muscle sample. The homogenate was divided into 6 aliquots and a standard mixture, containing 100 ng of each DAG species and 70 ng of each CER species was added before (n = 3) or after extraction procedure (n = 3). The recovery was examined by comparing the LC-MS/MS peak area of standard lipid species before and after extraction. The average recovery was calculated.2.6.2. Accuracy.

To assess assay accuracy, human muscle tissue was homogenized and aliquoted into 6 samples, to which known concentrations of DAG and CERs and IS were added. Precision of measurements is given as coefficient of variation (CV in %). Accuracy is displayed as measured concentration, corrected for endogenous DAG and CER concentrations, in percent of spiked concentration.

## 3. Statistical Analysis

Two-way ANOVA with Bonferroni correction were utilized for statistical analysis in GraphPad Prism software.

## 4. Results

### 4.1. Analysis of Diacylglycerols and Ceramides by LC-MS/MS

**Figure 1:**
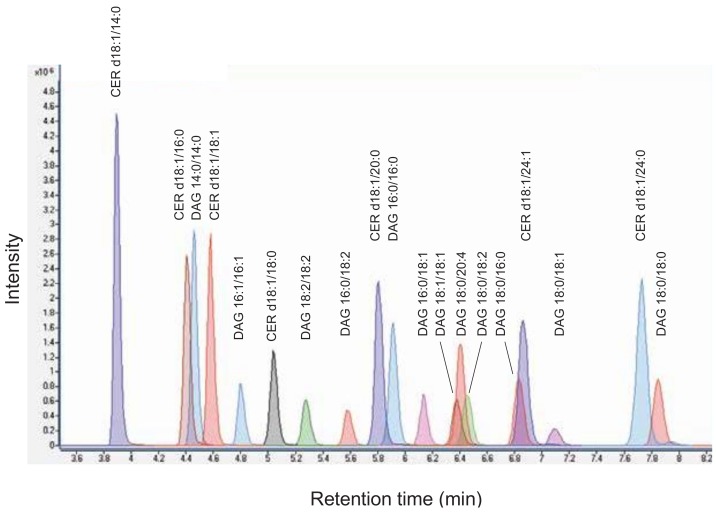
Chromatogram of diacylglycerol (DAG) and ceramide (CER) standard mixture. An overlay of multiple reaction monitoring (MRM) chromatograms of individual lipid species is shown.

To analyse the indicated DAG and CER species simultaneously by mass spectrometry, we applied ESI in positive ion mode and acquired product ion spectra. The collision energy (CE) for selected fragment ions was optimized. The limit of detection (LOD) was defined as signal to noise ratio of 3. LOD for DAG species ranged from 1.5 to 2.9 fmol, for CER species from 1.5 to 2.0 fmol on column (Table 1). The DAG and CER species were separated by liquid chromatography on a reverse-phase column using gradient elution within 10 min (Figure 1). 

Calibration curves for each analyte were generated by adding known concentrations of reference standards to sample buffer. Non-naturally occurring DAGs and CER were added as internal standard to compensate for variations in sample preparation and ionization efficiency. Excellent correlation of coefficients with R^2^ between 0.997 and 0.999 were found (Table 2, Supplementary Figure S1). Reproducibility of measurements of reference standards revealed both intra- and inter-day precision to be excellent, indicating highest precision of the MS-system (Supplementary Tables S1–S4). 

The extraction efficiency of the applied protocol was determined in human skeletal muscle homogenate by adding DAG and CER standard mixtures before and after extraction and comparing peak areas of individual lipid analytes. As shown in Table 3, mean recoveries of DAGs were between 73.6 and 115.1%, of CER between 81.3 and 118.3%.

Assay accuracy was determined by spiking known concentrations of analytes into human skeletal muscle homogenates. Accuracy for DAGs was found to be between 87.0 and 111.0%, for CERs between 83.6 and 123%. Precision was calculated in the above mentioned samples. The coefficient of variation (CV) was below 11.0% for all species analysed (Table 4).

### 4.2. Tissue Fractionation and LC-MS/MS Analysis of DAGs and CERs

**Figure 2:**
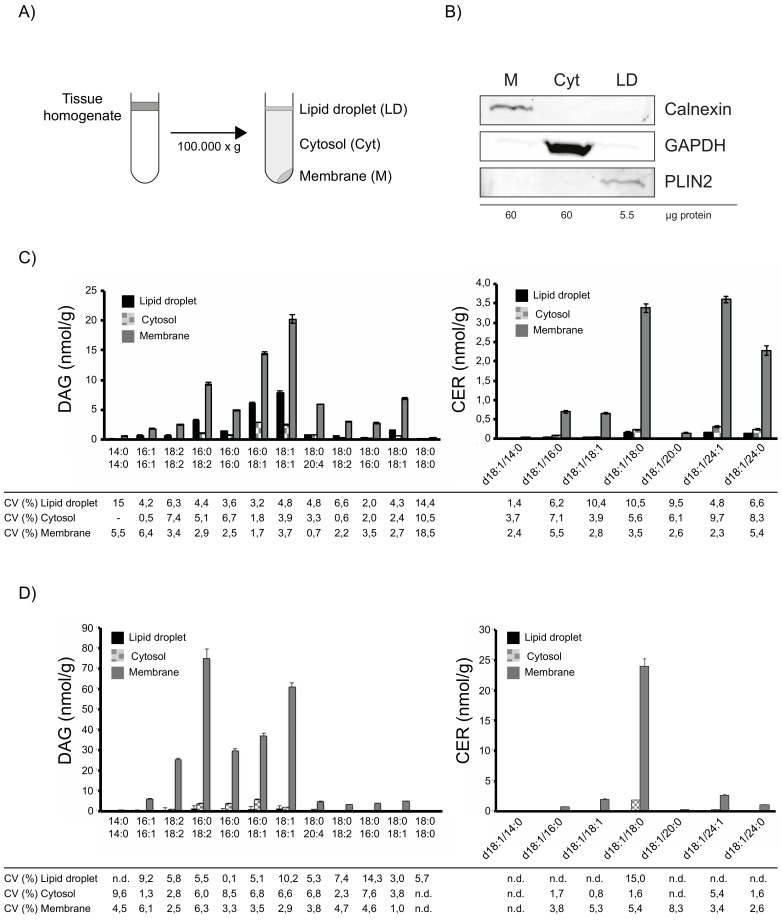
Subcellular fractionation and lipid analysis of human skeletal muscle. Schematic illustration of the fractionation protocol (**A**). Analysis of subcellular fractions obtained from human skeletal muscle. The protein concentration of the subcellular fractions after centrifugation was determined and the indicated amount was analysed by SDS-PAGE and western blotting using the indicated antibodies, Calnexin (ER); GAPDH (Cytosol); PLIN2 (LD) (**B**). Lipid mass spectrometry analysis of human muscle fractions. Human muscle sample was processed in triplicates and DAGs and CERs were analysed. Data are presented as mean ± SD. Correlation coefficient (CV) of individual lipid species in the indicated fraction is given in percent (**C**). Lipid mass spectrometry analysis of mouse muscle fractions. Mouse muscle sample was processed in triplicates and DAGs and CERs were analysed. Data are presented as mean ±SD. Correlation coefficient (CV) of individual lipid species in the indicated fraction is given in percent (**D**).

As previous studies addressing lipid-induced insulin resistance focused mainly on the accumulation of lipotoxins in membrane and cytosolic tissue fractions, we established a tissue fractionation protocol that allows separation of crude membrane, cytosolic and additional LD fractions using differential centrifugation, for subsequent simultaneous analysis of DAGs and CERs. Human muscle biopsy samples were homogenized, followed by differential centrifugation and the resulting fractions were collected (Figure 2A). The usage of a tube slicer after centrifugation increased purity of the collected floating LD layer and is thus highly recommended. Western Blotting analyses, using specific antibodies against marker proteins confirmed the purity of the obtained fractions (Figure 2B).

To test the feasibility of this method for subsequent lipid analyses, we analysed DAG and CER in subcellular fractions of human muscle samples. As shown in Figure 2C, DAGs were mainly found in membrane and LD fraction, whereas CERs were highly enriched in the membrane fraction. Precision of measurements in subcellular fractions was excellent with CVs below 10% for most DAGs and below 10.6% for all CERs. Similarly, measurements of DAG and CER in subcellular fractions of mouse muscle samples revealed excellent CVs below 10.3% for most DAGs and below 8.4% for most CERs. Together, these results demonstrate the applicability of the established method to analyse lipotoxic DAG and CER species in subcellular tissue fractions.

### 4.3. Multi-Tissue Lipid Analysis in a Mouse Model of NAFLD

NAFLD is characterized by the excessive accumulation of fat, mainly in liver. Additional studies highlight associated effects, for example, insulin resistance, on other tissues, such as skeletal muscle and heart [27,30,35,36]. Whereas a study in humans addressed the role of hepatic LDs as repository for lipotoxic DAG species in NAFL [37], their contribution to, for example, lipid-induced insulin resistance in other tissues has not been addressed in this context. Comprehensive studies revealed the accumulation of lipotoxic DAG species in membrane fractions of liver, skeletal muscle and heart in a mouse model of NAFLD [27,30]. In this model system, mice with adipose-specific overexpression of SREBP-1c develop NAFLD, secondary to lipid overflow from adipose tissue. In particular, mice at different ages were characterized by increased liver weight, increased hepatic triglycerides, portal and lobular inflammation, as well as hepatic and whole body insulin resistance [30,38]. To further analyse the role of LDs as repository for lipotoxic lipid species, we applied the newly established MS-approach to analyse the subcellular distribution of DAGs and CERs at multi-tissue level in greater detail in this mouse model of hepatic lipid overload with combined, hepatic, peripheral and whole body insulin resistance and controls. 

#### 4.3.1. Lipid Analysis of Liver

**Figure 3:**
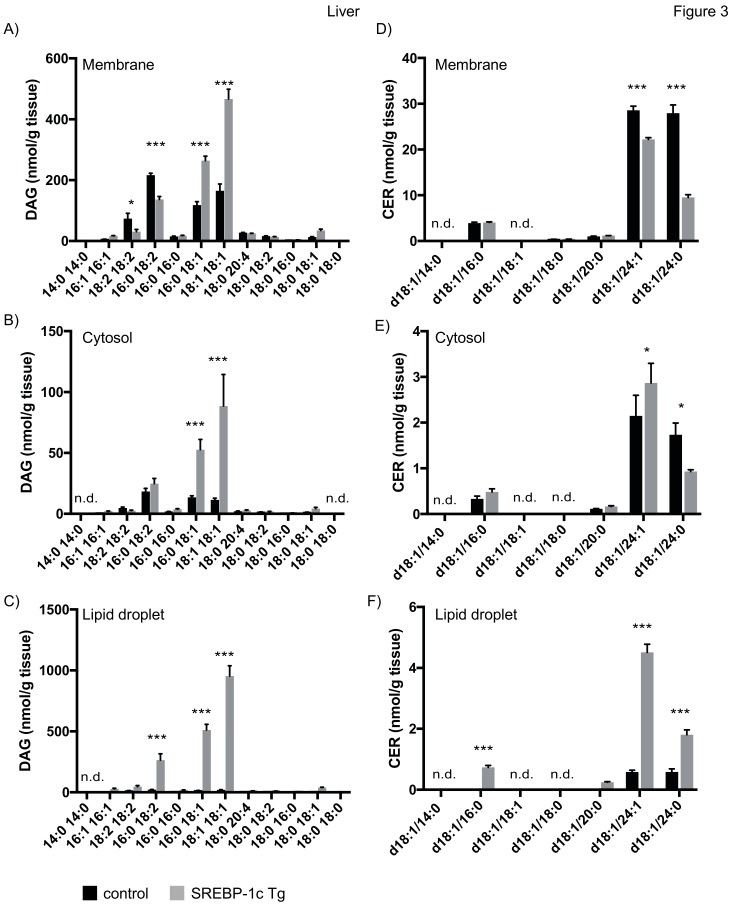
Mass spectrometry analysis of DAGs and CERs in liver tissue fractions. Liver tissue of SREBP-1c adipose-specific overexpressing transgenic (Tg) mice (SREBP-1c Tg) and control mice was subjected to subcellular fractionation. The indicated DAG species were analysed by LC-MS/MS in membrane (**A**), cytosolic (**B**) and LD fraction (**C**). The indicated CER species were analysed in membrane (**D**), cytosolic (**E**) and LD fraction (**F**). All data are presented as mean ± SEM (n = 6 per group). n.d., not detected; * *p* < 0.05, *** *p* < 0.001; 2-way ANOVA with Bonferroni correction.

Consistent with previous results, we observed a strong increase in membrane DAG 18:1/18:1 in liver of NAFLD mice [30]. In addition, our studies revealed an increase in DAG 16:0/18:1 and a decrease in DAG 16:0/18:2, DAG 18:2/18:2 in membranes (Figure 3A). Analysis of cytosolic fractions revealed an increase of DAG 16:0/18:1 and DAG 18:1/18:1 in NAFLD mice (Figure 3B). Interestingly, we observed an even stronger increase in DAG 16:0/18:2, DAG 16:0/18:1 and DAG 18:1/18:1 in the LD fraction of these mice (Figure 3C). Whereas NAFLD led to an approx. 2.5fold increase of the above mentioned DAG species in the membrane fraction, an approximately 500–1000 fold increase in the LD fraction was observed. Intriguingly, the concentration of DAG 18:1/18:1 was twofold higher in the LD fraction compared to membrane. We detected CER d18:1/24:1 and d18:1/24:0 as major CER species in liver. These species decreased in membranes of NAFLD mice, in line with a decrease of these species in a previous study (Figure 3D) [30]. In contrast, we observed an increase in d18:1/24:1 and d18:1/24:0 in LDs of NAFLD mice (Figure 3F).

#### 4.3.2. Lipid Analysis of Skeletal Muscle

**Figure 4:**
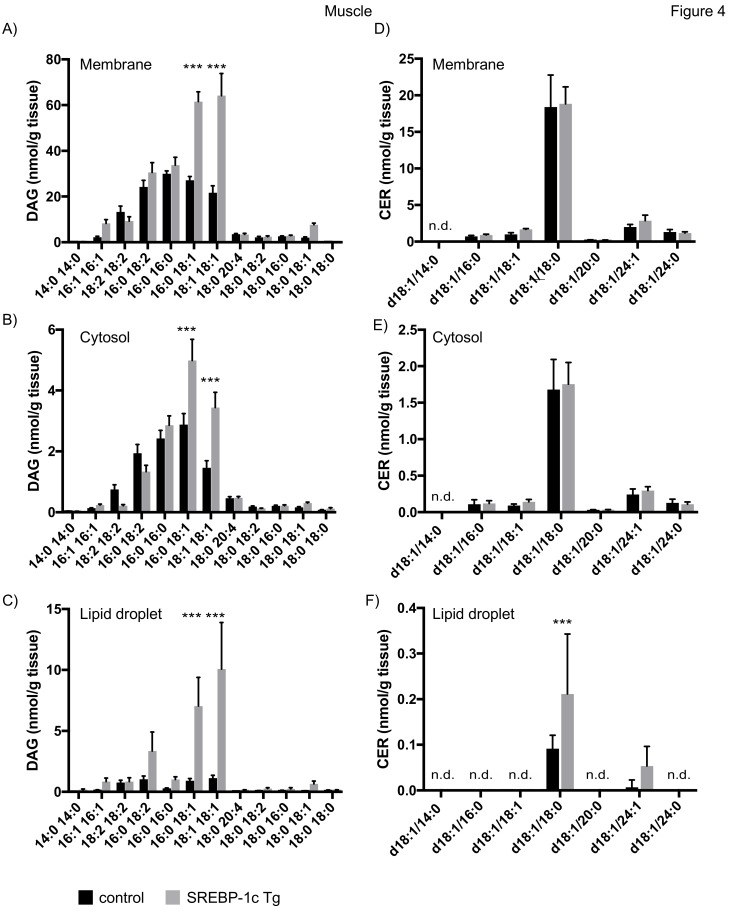
Mass spectrometry analysis of DAGs and CERs in skeletal muscle tissue fractions. Muscle tissue of SREBP-1c adipose-specific overexpressing transgenic (Tg) mice (SREBP-1c Tg) and control mice was subjected to subcellular fractionation. The indicated DAG species were analysed by LC-MS/MS in membrane (**A**), cytosolic (**B**) and lipid droplet fraction (**C**). The indicated CER species were analysed in membrane (**D**), cytosolic (**E**) and LD fraction (**F**). All data are presented as mean ± SEM (n = 6 per group). n.d., not detected; *** *p* < 0.001; 2-way ANOVA with Bonferroni correction.

Analysis of subcellular fractions of skeletal muscle samples revealed an increase of DAG 16:0/18:1 and DAG 18:1/18:1 in membrane and cytosolic fraction of NAFLD mice (Figure 4A,B), consistent with previous results [30]. However, here we show a 5–10 fold increase in these lipid species in the LD fraction (Figure 4C). In contrast to liver samples, CER d18:0/18:0 was the major CER species detected in muscle. No changes in CER concentration were observed in membrane and cytosolic fraction, whereas an increase of CER d18:1/18:0 was observed in LD fractions in NAFLD mice (Figure 4D–F).

#### 4.3.3. Lipid Analysis of Heart

**Figure 5:**
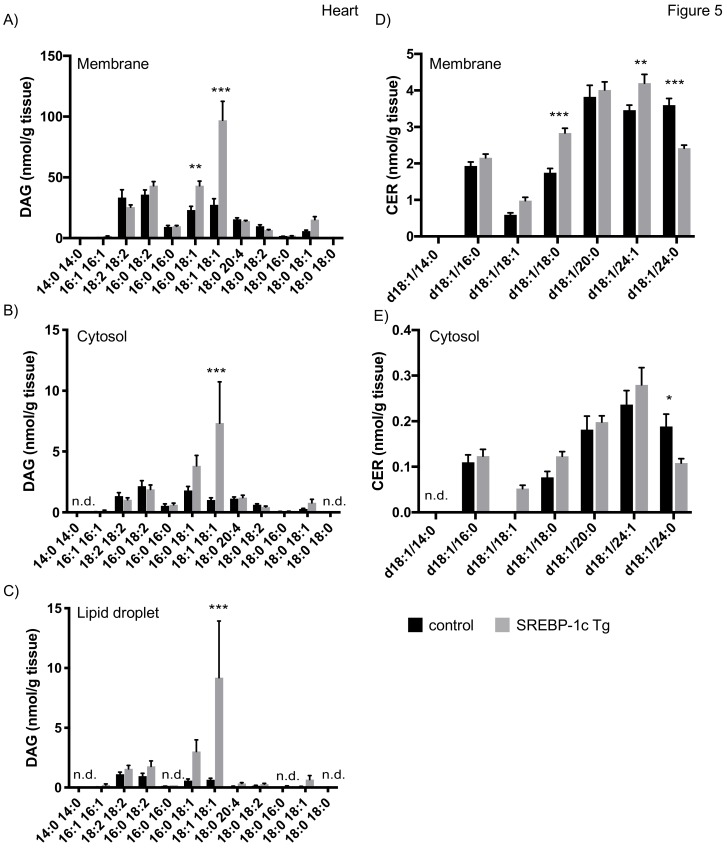
Mass spectrometry analysis of DAGs and CERs in heart tissue fractions. Heart tissue of SREBP-1c adipose-specific overexpressing transgenic (Tg) mice (SREBP-1c Tg) and control mice was subjected to subcellular fractionation. The indicated DAG species were analysed by LC-MS/MS in membrane (**A**), cytosolic (**B**) and LD fraction (**C**). The indicated CER species were analysed in membrane (**D**) and cytosolic fraction (**E**). All data are presented as mean ± SEM (n = 6 per group). n.d., not detected; * *p* < 0.05, ** *p* < 0.01, *** *p* < 0.001; 2-way ANOVA with Bonferroni correction.

In heart of mice with NAFLD, we observed an increase of DAG 18:1/18:1 in membrane and cytosolic fraction, consistent with previous results [27]. In addition, we report an increase of DAG 16:0/18:1 in membrane fractions (Figure 5A,B). Our analyses furthermore revealed that both species are increased approximately 5–10 fold in LDs of NAFLD mice (Figure 5C). No data on CER concentration in the heart have been reported for this model in the literature. We here show that, in contrast to liver and skeletal muscle, no prominent CER species were detected but rather a uniform species distribution at lower concentrations was observed (Figure 5A,B). CER d18:1/18:0 and d18:1/24:1 were increased in membrane fraction and CER d18:1/24:0 was decreased in both, membrane and cytosolic fraction (Figure 5D,E). CERs were rarely detected in LD fraction of heart samples, thereby preventing a representative analysis.

In summary, the newly established targeted lipidomics approach allowed us to conduct a detailed multi-tissue analysis of the subcellular distribution of lipotoxic DAGs and CERs in a mouse model of NAFLD, revealing a prominent role for LDs in sequestering oleic acid-containing DAG and specific CER species. 

## 5. Discussion

The dysregulation of lipid metabolism both, at the cellular and tissue level, is a hallmark of various metabolic disorders [2]. Lipid overload, as observed in obesity and associated diseases, such as T2D and NAFLD, leads to the accumulation of specific lipotoxic lipid species, such as DAGs and CERs, which were shown to induce insulin resistance [18,39]. Their lipotoxic properties are most likely influenced by their subcellular localization. However, detailed analyses on the subcellular distribution of these lipids in multiple tissues are mainly hampered by biochemically challenging sample preparation and high requirements regarding lipid analysis using mass spectrometry. Here, we present a LC-MS/MS approach to simultaneously analyse lipotoxic DAG and CER species in subcellular fractions, in particular pure LDs, of tissue samples and apply it to a mouse model of NAFLD, highlighting the role of LDs in multiple tissues as potential repository for these lipids. 

Previous studies on lipid-induced insulin resistance in various tissues, such as liver and muscle, provide a variety of information regarding the subcellular distribution of lipotoxic DAGs and CERs, depending on the fraction protocol used. To date, most studies separated crude membranes, containing various organelle membranes, such as ER, mitochondria and plasma membrane and cytosolic tissue fractions. As these studies used differential centrifugation to separate fractions, the cytosolic fraction contains the floating LD layer [29]. Therefore, results from these studies do not specifically address the role of LDs in processing these disease-relevant lipids. Of note, recent studies applied improved fraction protocols which allow further separation of cellular membranes [29]. Using a sophisticated fractionation protocol in combination with comprehensive proteomics analyses, Mann and colleagues obtained various membrane fractions and seemingly pure LD fraction. However, data on the lipid composition of these fractions are not available [32]. Here, we improved currently applied differential centrifugation protocols to fractionate tissue homogenates. The floating LD fraction was collected using a tube slicer, thereby improving purity of the fraction and prevent cross-contamination with cytosol. As an example, we applied this method to human skeletal muscle samples and obtained highly pure membrane, cytosolic and LD fractions allowing us to specifically address the role of LDs in the above mentioned metabolic disorders.

Lipids were extracted from samples using a well-established extraction protocol and separated using standard reverse-phase chromatography prior to analysis via mass spectrometry, making this method an easy to adapt approach. Calibration was performed by adding naturally occurring lipid species and IS to sample buffer prior to extraction. The MS parameters were optimized and assay characteristics were determined. Overall, the established method is characterized by excellent sensitivity, precision and accuracy.

We applied our method to an established mouse model of NAFLD. Mice with adipose tissue specific overexpression of sterol-element-binding protein 1c (SREBP-1c) show a marked loss of adipose tissue, probably due to decreased lipogenesis and impaired insulin-mediated suppression of lipolysis, leading to a redirection of lipid flux towards peripheral tissues [39]. The secondary deposition of lipids in liver, muscle and heart was shown to trigger insulin resistance via DAG-mediated activation of nPKC isoforms [27,30]. As these studies analysed DAGs and CERs in membrane and cytosolic fraction we used the newly established method to complete these investigations by analysing the role of LDs as potential repository for lipotoxic lipid species in this mouse model. Consistent with the previous studies, our data reveal an increase of oleic acid containing DAGs, mainly 18:1/18:1 in membrane and cytosolic fractions of liver, muscle and heart. In addition, the accumulation of DAG 16:0/18:1 was observed, being in line with the concept that unsaturated DAG species are efficient activators of nPKCs [40]. Importantly, we now show a strong increase in DAG 16:0/18:1 and 18:1/18:1 in LDs in liver, muscle and heart of NAFLD mice. Remarkably, the concentration of these DAG species in hepatic LDs exceeded the levels in the membrane fraction approximately 2fold in NAFLD mice. Overall, NAFLD lead to 2-3 fold increase of the described DAG species in membrane fractions of liver, muscle and heart, whereas an increase of approximately 5–10 fold was observed in LDs of muscle and heart and up to 1000fold in hepatic LDs. It is important to note that the strong increase in DAGs in LDs most likely leads to a highly localized and spatially restricted accumulation of bioactive lipids, as these organelles are much more defined as the broadly distributed membrane network spanning the cell. 

How can one integrate these results into the current working model of DAG and nPKC-mediated insulin resistance? It is generally believed that the accumulation of specific DAGs in membranes upon excessive lipid loading leads to the recruitment and activation of nPKC isoforms [41]. DAGs are mainly synthesized by enzymes localizing to the ER membrane and the LDs. Our findings that accumulated DAG species also accumulate in LDs therefore raises the question whether DAGs, stored in this compartment, remain bioactive and recruit and activate nPKCs or whether it serves as a protective organelle, sequestering and shielding otherwise lipotoxic lipid species. Interestingly, studies focusing on NAFLD in mice and humans revealed that also nPKCε localizes to LDs [32,37]. However, whether nPKCε is active when recruited to this organelle is currently unclear.

Our data on CER concentration in liver and muscle are in line with previous results [30]. Importantly, our approach now reveals an increase of d18:1/24:1 and d18:1/24:0 in hepatic LDs and d18:1/18:0 in muscle LDs of NAFLD mice. Of note, a recent study showed that CERs can be converted to acylceramides in LDs. In fact, high-fat diet in mice induced the accumulation of acylceramides in hepatic LDs, which therefore serve as storage units for ceramides upon excessive lipid loading [42]. Similarly, the liver of NAFLD mice is exposed to excessive lipid delivery. It is therefore conceivable that the accumulated CER species in hepatic LDs, observed in this study, serve as substrate for the generation of acylceramides on LDs.

The results presented here suggest that LDs are important cellular organelles contributing to the pathological mechanisms underlying fatty liver disease. Despite their function as repository for lipotoxic lipid intermediates it remains unclear how storage of lipotoxins in LDs affects disease-related processes. It is tempting to speculate that regulating the lipid content of LDs or LD storage capacity in general, as potential treatment approach might alleviate the disease pattern of NAFLD. However, despite the identification and characterization of lipotoxin-regulating reagents, for example, diglyceride acyltransferase (DGAT)-inhibitors, additional research is required to validate their applicability as therapeutic tool.

Taken together, we established an easy to apply LC-MS/MS based method to analyse lipotoxic DAG and CER species in subcellular fractions, that is, pure LDs of tissue samples. Applying this method to a mouse model of NAFLD reveals a prominent role for LDs as repository of lipotoxic DAG species. This method therefore presents a valuable tool to analyse subcellular dynamics of lipotoxins underlying the pathogenesis of insulin resistance in metabolic disorders.

## Figures and Tables

**Table 1:** Mass spectrometry (MS) parameter and limit of detection (LOD) of lipid species.

Lipid Class	Lipid Species	Ion	MRM	CE (V)	RT (min)	LOD (fmol) On Column
DAG	14:0 14:0	[M + NH4]^+^	530.2 → 285.0	16	4.50	2.0
DAG	16:1 16:1	[M + NH4]^+^	582.2 → 311.1	26	4.84	1.5
DAG	18:2 18:2	[M + NH4]^+^	634.3 → 337.2	26	5.30	1.6
DAG	16:0 18:2	[M + NH4]^+^	610.2 → 313.2	26	5.63	1.7
DAG	16:0 16:0	[M + NH4]^+^	586.2 → 313.0	16	5.96	1.8
DAG	16:0 18:1	[M + NH4]^+^	612.1 → 339.1	30	6.20	1.7
DAG	18:1 18:1	[M + NH4]^+^	638.2 → 339.1	28	6.43	1.6
DAG	18:0 20:4	[M + NH4]^+^	662.2 → 341.3	20	6.45	1.6
DAG	18:0 18:2	[M + NH4]^+^	638.1 → 341.0	24	6.50	1.6
DAG	18:0 16:0	[M + NH4]^+^	614.2 → 313.1	24	6.89	1.7
DAG	18:0 18:1	[M + NH4]^+^	640.2 → 341.2	30	7.15	1.6
DAG	18:0 18:0	[M + NH4]^+^	642.1 → 341.0	28	7.92	1.6
CER	d18:1/14:0	[M + H]^+^	510.3 → 264.0	34	3.93	2.0
CER	d18:1/16:0	[M + H]^+^	538.2 → 264.0	32	4.44	1.9
CER	d18:1/18:1	[M + H]^+^	564.2 → 264.0	36	4.61	1.8
CER	d18:1/18:0	[M + H]^+^	566.1 → 264.1	30	5.07	1.8
CER	d18:1/20:0	[M + H]^+^	594.2 → 264.0	36	5.86	1.7
CER	d18:1/24:1	[M + H]^+^	648.3 → 264.1	34	6.91	1.5
CER	d18:1/24:0	[M + H]^+^	650.5 → 264.0	34	7.78	1.5

MRM, multiple reaction monitoring; CE, collision energy; RT, retention time; LOD, limit of detection.

**Table 2:** Calibration data of the indicated lipid species.

Lipid Class	Lipid Species	Calibration Range (ng)	Calibration Range (pmol)	IS Added (ng; pmol)	Correlation Coefficient (mean ± SD)
DAG	14:0 14:0	1–1000	1.95–1950	500; 830	0.999 ± 0.001
DAG	16:1 16:1	1–1000	1.77–1770	500; 830	0.998 ± 0.001
DAG	18:2 18:2	1–1000	1.62–1621	500; 830	0.998 ± 0.001
DAG	16:0 18:2	1–1000	1.69–1687	500; 830	0.998 ± 0.001
DAG	16:0 16:0	1–1000	1.76–1758	500; 830	0.999 ± 0.000
DAG	16:0 18:1	1–1000	1.68–1681	500; 830	0.998 ± 0.000
DAG	18:1 18:1	1–1000	1.61–1610	500; 830	0.999 ± 0.000
DAG	18:0 20:4	1–1000	1.55–1550	500; 830	0.998 ± 0.000
DAG	18:0 18:2	1–1000	1.61–1610	500; 830	0.998 ± 0.000
DAG	18:0 16:0	1–1000	1.68–1675	500; 830	0.999 ± 0.001
DAG	18:0 18:1	1–1000	1.61–1605	500; 830	0.997 ± 0.001
DAG	18:0 18:0	1–1000	1.77–1585	500; 830	0.999 ± 0.001
CER	d18:1/14:0	1–200	1.96–392	100; 181	0.997 ± 0.002
CER	d18:1/16:0	1–200	1.86–372	100; 181	0.999 ± 0.001
CER	d18:1/18:1	1–200	1.77–355	100; 181	0.999 ± 0.000
CER	d18:1/18:0	1–200	1.77–353	100; 181	0.995 ± 0.003
CER	d18:1/20:0	1–200	1.68–337	100; 181	0.997 ± 0.002
CER	d18:1/24:1	1–200	1.54–309	100; 181	0.997 ± 0.002
CER	d18:1/24:0	1–200	1.54–308	100; 181	0.997 ± 0.001

8-point calibration curves were generated by plotting ratio of analyte to internal standard (IS) against the concentration of the added reference standard. The data are presented as mean ± SD (n = 3).

**Table 3:** Recovery.

Lipid Class	Lipid Species	Spiked Amount (ng; pmol)	Recovery (%)
DAG	14:0 14:0	100; 195	73.6 ± 3.4
DAG	16:1 16:1	100; 177	86.7 ± 2.7
DAG	18:2 18:2	100; 162	88.1 ± 4.2
DAG	16:0 18:2	100; 169	91.7 ± 0.9
DAG	16:0 16:0	100; 176	105.2 ± 3.5
DAG	16:0 18:1	100; 168	105.3 ± 3.2
DAG	18:1 18:1	100; 161	115.1 ± 1.0
DAG	18:0 20:4	100; 155	97.0 ± 4.2
DAG	18:0 18:2	100; 161	97.1 ± 2.7
DAG	18:0 16:0	100; 168	79.7 ± 0.5
DAG	18:0 18:1	100; 161	83.0 ± 1.0
DAG	18:0 18:0	100; 160	72.4 ± 5.3
CER	d18:1/14:0	70; 137	97.0 ± 4.3
CER	d18:1/16:0	70; 130	91.0 ± 2.9
CER	d18:1/18:1	70; 124	87.2 ± 2.8
CER	d18:1/18:0	70; 124	81.3 ± 2.5
CER	d18:1/20:0	70; 118	118.3 ± 3.3
CER	d18:1/24:1	70; 108	89.2 ± 2.3
CER	d18:1/24:0	70; 108	118.3 ± 0.6

Homogenate of human muscle sample was aliquoted and standard mixture containing 100 ng of each DAG species and 70 ng of each CER species was added before (n = 3) or after extraction procedure (n = 3). The recovery was examined by comparing the LC-MS/MS peak area of standard lipid species before and after extraction. The average recovery ± SD is shown.

**Table 4:** Precision and Accuracy.

Lipid Class	Lipid Species	Spiked Amount (ng; pmol)	CV (%)	Accuracy (%)
DAG	14:0 14:0	125; 244	6.4	109 ± 3.6
DAG	16:1 16:1	125; 221	9.0	108 ± 4.5
DAG	18:2 18:2	125; 203	11.0	102 ± 5.9
DAG	16:0 18:2	125; 211	8.6	87.0 ± 8.1
DAG	16:0 16:0	125; 220	7.4	106 ± 5.4
DAG	16:0 18:1	125; 210	6.3	111 ± 10.3
DAG	18:1 18:1	125; 201	8.3	103 ± 14.8
DAG	18:0 20:4	125; 194	8.3	114 ± 5.7
DAG	18:0 18:2	125; 201	9.0	111 ± 6.2
DAG	18:0 16:0	125; 209	6.3	106 ± 4.0
DAG	18:0 18:1	125; 201	5.4	98.9 ± 6.0
DAG	18:0 18:0	125; 201	1.3	95.0 ± 0.6
CER	d18:1/14:0	100; 196	4.9	83.6 ± 1.7
CER	d18:1/16:0	100; 186	4.1	107 ± 2.1
CER	d18:1/18:1	100; 177	5.4	96.6 ± 2.2
CER	d18:1/18:0	100; 177	4.1	116 ± 2.4
CER	d18:1/20:0	100; 168	7.7	123 ± 4.3
CER	d18:1/24:1	100; 154	9.0	110 ± 5.2
CER	d18:1/24:0	100; 154	7.2	118 ± 4.3

A pool of human muscle homogenate was aliquoted and individual aliquots were spiked with the indicated amount of lipid standard (n = 5–6). Precision is given as coefficient of variation (CV in %). Accuracy is reported as the mean ± SD of the assayed concentration, corrected for endogenous DAG and CER concentrations, in percent of the spiked concentration.

## Supplementary Materials

The following are available online at https://www.mdpi.com/2073-4409/8/3/277/s1, Figure S1: title, Table S1: title.
