# Supplementary material for: A New Targeted Lipidomics Approach Reveals Lipid Droplets in Liver, Muscle and Heart as a Repository for Diacylglycerol and Ceramide Species in Non-Alcoholic Fatty Liver
Source: Cells. 2019 Mar 22;8(3):277. doi: 10.3390/cells8030277 (PMC6468791; doi:10.3390/cells8030277)
Supplement: Supplementary file 1 [file cells-08-00277-s001.pdf]

Supplement

DAG 18:1 18:1

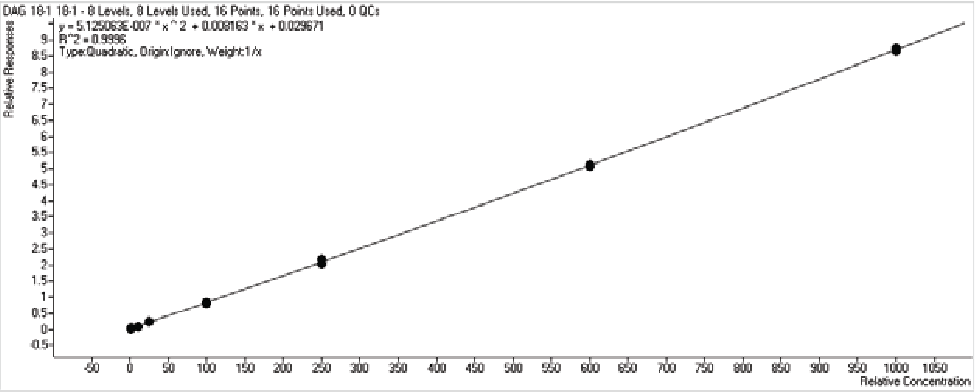

CER d18:1/18:0

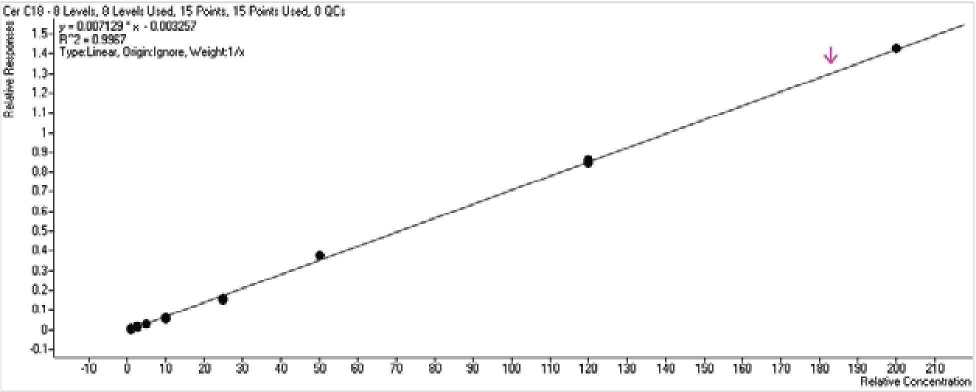

Figure 1: Representative concentration standard curves for DAG 18:1/18:1 and CER d18:1/18:0

Table 1. Intraday precision of DAG reference standards

DAG	Nominal (pmol)	Intraday (pmol ± SD)	CV (%)	Accuracy (%)
**14:0 14:0**	5.9	6,04 ± 0.5	7.8	103 ± 8.1
	244	251 ± 8.2	3.3	103 ± 3.4
	488	500 ± 31.8	6.4	103 ± 6.5
**16:1 16:1**	5.3	5.54 ± 0.3	5.6	104 ± 5.9
	221	238 ± 8.8	3.7	108 ± 4.0
	443	476 ± 11.9	2.5	108 ± 2.7
**18:2 18:2**	4.9	4.76 ± 0.4	8.3	98.0 ± 8.2
	203	191 ± 3.9	2.1	94.3 ± 1.9
	405	389 ± 17.4	4.5	96.1 ± 4.3
**16:0 18:2**	5.1	4.87 ± 0.5	11	100 ± 10.6
	211	183 ± 6.5	3.5	90.1 ± 3.2
	422	361 ± 10.9	3.0	89.2 ± 2.7
**16:0 16:0**	5.27	5.47 ± 0.5	9.1	104 ± 9.4
	220	203 ± 7.4	3.6	92.3 ± 3.4
	439	420 ± 24.8	5.9	95.6 ± 5.6
**16:0 18:1**	5.0	5.18 ± 0.4	7.9	103 ± 8.1

	210	226 ± 8.9	3.9	108 ± 4.2
	420	445 ± 13.8	3.1	106 ± 3.3
**18:1 18:1**	4.8	4.92 ± 0.2	4.6	102 ± 4.7
	201	216 ± 7.5	3.5	107 ± 3.7
	403	419 ± 17.6	4.2	104 ± 4.4
**18:0 20:4**	4.7	4.75 ± 0.5	10	102 ± 10.4
	194	203 ± 9.0	4.4	105 ± 4.6
	388	369 ± 20.5	5.5	95.2 ± 5.3
**18:0 18:2**	4.8	4.67 ± 0.4	8.5	96.6 ± 8.3
	201	203 ± 3.9	1.9	101 ± 1.9
	403	401 ± 13.5	3.4	99.7 ± 3.4
**18:0 16:0**	5.0	5.24 ± 0.26	5.0	104 ± 5.3
	209	218 ± 6.9	3.2	104 ± 3.3
	419	434 ± 22.7	5.2	104 ± 5.4
**18:0 18:1**	4.8	4.86 ± 0.4	7.7	101 ± 7.7
	201	201 ± 9.7	4.8	100 ± 4.9
	401	389 ± 21.4	5.5	5.3 ± 96.9
**18:0 18:0**	4.8	5.18 ± 0.3	5.7	108 ± 6.1
	200	183 ± 7.7	4.2	91.3 ± 3.8
	400	375 ± 24.0	6.4	93.7 ± 6.0

The indicated amounts of DAG species, corresponding to 3, 125 and 250 ng, were processed and analyzed for intraday precision (n=5-8) Data are presented as mean ± SD. Accuracy is reported as the mean ± SD of the assayed concentration, in percent of nominal concentration. CV, Coefficient of variation.

Table 2. Intraday precision of CER reference standards

**CER**	**Nominal (pmol)**	**Intraday (pmol ± SD)**	**CV (%)**	**Accuracy (%)**
**d18:1/14:0**	5.9	5.64 ± 0.3	5.0	95.8 ± 4.8
	196	171 ± 3.7	2.2	87.4 ± 1.9
	343	317 ± 16.5	5.2	92.2 ± 4.8
**d18:1/16:0**	5.6	6.06 ± 0.2	3.7	109 ± 4.0
	186	194 ± 8.3	4.3	104 ± 4.5
	325	353 ± 12.9	3.7	108 ± 4.0
**d18:1/18:1**	5.3	5.05 ± 0.3	5.9	94.9 ± 5.6
	177	164 ± 8.5	5.2	92.5± 4.8
	310	289 ± 13.4	4.6	93.2 ± 4.3
**d18:1/18:0**	5.3	5.35 ± 0.3	6.3	101 ± 6.4
	177	180 ± 12.8	7.1	102 ± 7.2
	309	322 ± 20.0	6.2	104 ± 6.5
**d18:1/20:0**	5.1	5.46 ± 0.3	5.0	108 ± 5.4
	168	169 ± 10.9	6.4	101 ± 6.5
	295	306 ± 7.4	2.4	104 ± 2.5
**d18:1/24:1**	4.6	4.74 ± 0.3	6.7	102 ± 6.8
	154	158 ± 7.6	4.8	102 ± 4.9
	270	268 ± 8.2	3.1	99.2 ± 3.0

**d18:1/24:0**	4.6	4.38 ± 0.2	5.1	94.8 ± 4.8
	154	144 ± 10.7	7.4	93.6 ± 6.9
	269	280 ± 22.1	7.9	104 ± 8.2

The indicated amounts of CER species, corresponding to 3, 125 and 250 ng, were processed and analyzed for intraday precision (n=5-8) Data are presented as mean ± SD. Accuracy is reported as the mean ± SD of the assayed concentration, in percent of nominal concentration. CV, Coefficient of variation.

Table 3. Interday precision of DAG reference standards

DAG	Nominal (pmol)	Interday (pmol ± SD)	CV (%)	Accuracy (%)
14:0 14:0	1,95	2,36 ± 0.2	10,3	121 ± 12.7
	4,88	4,65 ± 0.4	7,6	95,1 ± 6.9
	19,5	18,2 ± 0.6	3,2	93,1 ± 2.9
	48,8	50,0 ± 0.6	1,1	102 ± 1.3
	195	182 ± 2.4	1,3	93,6 ± 1.2
	488	502 ± 10.7	2,1	103 ± 1.7
	1170	1168 ± 18,4	1,6	100 ± 1.8
	1950	1949 ± 7,4	0,4	100 ± 0.4
16:1 16:1	1,77	2,05 ± 0.1	5,7	116 ± 6.6
	4,43	4,54 ± 0.2	3,3	103 ± 3.4
	17,7	15,94 ± 0.9	5,6	90,0 ± 5.0
	44,3	44,1 ± 1.5	3,3	100 ± 3.3
	177	159,0 ± 4.2	2,7	93,5 ± 4.2
	443	444 ± 8.9	2,0	100 ± 2.0
	1062	1094 ± 25.7	2,4	103 ± 2.6
	1770	1755 ± 11.6	0,7	99,1 ± 0.7
18:2 18:2	1,62	1,97 ± 0.1	7,0	119 ± 9.5
	4,05	4,01 ± 0.1	1,5	101 ± 1.8
	16,2	14,8 ± 0.4	2,5	91,4 ± 2.7
	40,5	39,1 ± 1.8	4,6	96,8 ± 4.4
	162	145 ± 5.5	3,8	89,2 ± 3.3
	405	408 ± 3.2	0,8	100 ± 0.6
	972	1001 ± 13.1	1,3	103 ± 1.4
	1621	1607 ± 6.6	0,4	99,0 ± 0.4
16:0 18:2	1,69	2,00 ± 0.1	3,8	119 ± 4.5
	4,22	4,35 ± 0.2	3,7	103 ± 3.9
	16,9	15,4 ± 0.4	2,6	91,15 ± 2.4
	42,2	40,4 ± 0.7	1,7	95,9 ± 1.7
	169	148 ± 2.6	1,7	87,8 ± 1.5
	422	423 ± 0.6	0,1	100 ± 0.1
	1012	1054 ± 7.5	0,7	104 ± 1.1
	1687	1665 ± 3.8	0,2	98,7 ± 0.2
16:0 16:0	1,76	1,82 ± 0.1	7,9	104 ± 8.2
	4,39	4,59 ± 0.3	6,1	105 ± 6.3
	17,6	17,4 ± 1.4	7,8	99,31 ± 7.9
	43,9	44,7 ± 1.0	2,2	102 ± 2.1
	176	158 ± 1.0	0,6	89,9 ± 0.6
	439	432 ± 0.9	0,2	98,3 ± 0.2
	1055	1092 ± 2.1	0,2	104 ± 0.5

	1758	1742 ± 0.6	0,0	99,1 ± 0.0
16:0 18:1	1,68	1,98 ± 0.1	2,3	118 ± 2.7
	4,20	4,37 ± 0.1	2,5	104 ± 2.8
	16,8	14,9 ± 0.9	6,1	88,5 ± 5.4
	42,0	40,5 ± 1.5	3,7	96,3 ± 3.5
	168	146 ± 1.4	0,9	86,6 ± 0.8
	420	422 ± 6.1	1,4	100 ± 1.4
	1008	1045 ± 8.9	0,9	104 ± 0.7
	1681	1663 ± 3.4	0,2	99 ± 0.2
18:1 18:1	1,61	1,74 ± 0.0	1,5	108 ± 1.6
	4,03	3,86 ± 0.0	0,8	96,0 ± 0.8
	16,1	15,1 ± 1.0	6,7	93,7 ± 6.3
	40,3	41,9 ± 1.6	3,7	104 ± 3.8
	161	156 ± 3.3	2,1	96,7 ± 2.1
	403	409 ± 4.2	1,0	102 ± 1.1
	966	964 ± 1.1	0,1	100 ± 0.2
	1610	1611 ± 1.0	0,1	100 ± 0.0
18:0 20:4	1,55	2,06 ± 0.1	3,5	133 ± 4.6
	3,88	3,77 ± 0.1	1,2	97,1 ± 1.3
	15,5	13,0 ± 0.6	4,4	83,5 ± 3.7
	38,8	36,6 ± 0.8	2,1	94,4 ± 2.1
	155	136 ± 2.1	1,5	87,5 ± 1.4
	388	398 ± 3.2	0,8	103 ± 0.9
	930	957 ± 10.8	1,1	103 ± 1.3
	1550	1536 ± 5.1	0,3	99,1 ± 0.3
18:0 18:2	1,61	2,01 ± 0.0	1,2	125 ± 1.5
	4,03	3,90 ± 0.1	1,2	96,8 ± 1.1
	16,1	13,6 ± 0.7	4,9	84,5 ± 4.1
	40,3	37,7 ± 1.3	3,3	93,6 ± 3.0
	161	144 ± 1.5	1,0	89,3 ± 0.9
	403	412 ± 1.8	0,4	102 ± 0.5
	966	989 ± 4.4	0,4	102 ± 0.6
	1610	1598 ± 2.0	0,1	99,2 ± 0.1
18:0 16:0	1,68	1,82 ± 0.2	9,8	108 ± 10.6
	4,19	4,20 ± 0.2	5,4	100 ± 5.4
	16,8	16,4 ± 1.2	7,3	97,8 ± 7.1
	41,9	42,6 ± 2.6	6,0	102 ± 6.3
	168	153 ± 3.8	2,5	91,1 ± 2.3
	419	413 ± 3.6	0,9	98,7 ± 0.9
	1005	1037 ± 12.4	1,2	103 ± 1.2
	1675	1661 ± 5.3	0,3	99,1 ± 0.3
18:0 18:1	1,61	1,86 ± 0.1	3,0	116 ± 3.4
	4,01	4,16 ± 0.1	2,3	103 ± 2.5
	16,1	15,0 ± 0.4	2,4	93,7 ± 2.3
	40,1	39,2 ± 1.0	2,5	97,4 ± 2.3
	161	140 ± 5.2	3,7	87,2 ± 3.3
	401	396 ± 1.9	0,5	98,7 ± 0.4
	963	1016 ± 8.5	0,8	105 ± 0.6
	1605	1579 ± 4.3	0,3	98 ± 0.3
18:0 18:0	1,60	1,77 ± 0.1	5,0	110 ± 5.4
	4,00	4,15 ± 0.1	2,8	104 ± 2.8

16,0	14,8 ± 0.3	2,1	92,5 ± 1.9
40,0	41,4 ± 1.8	4,4	104 ± 4.7
160	142 ± 3.3	2,3	88,6 ± 2.0
400	395 ± 4.2	1,1	98,8 ± 1.2
960	995 ± 8.8	0,9	104 ± 1.2
1600	1585 ± 3.7	0,2	99,1 ± 0.2

The indicated amounts of lipids were processed and analyzed at three consecutive days. Data are presented as mean ± SD. Accuracy is reported as the mean ± SD of the assayed concentration, in percent of nominal concentration. CV, Coefficient of variation.

Table 4. Interday precision of CER reference standards

Ceramide	Nominal (pmol)	Interday (pmol ± SD)	CV (%)	Accuracy (%)
d18:1/14:0	1,96	2,13 ± 0.2	7,4	109 ± 8.0
	4,90	4,78 ± 0.1	1,9	98,0 ± 1.4
	9,81	9,11 ± 0.4	4,0	92,9 ± 3.9
	19,6	18,5 ± 0.2	1,0	94,4 ± 0.9
	49,0	49,8 ± 3.3	6,6	101 ± 6.7
	98,1	107 ± 4.5	4,3	109 ± 4.9
	235	240 ± 6.0	2,5	102 ± 2.4
	392	380 ± 11.8	3,1	96,8 ± 3.0
d18:1/16:0	1,86	2,17 ± 0.1	2,5	117 ± 2.8
	4,65	4,62 ± 0.2	3,4	99,4 ± 3.5
	9,30	8,53 ± 0.2	2,3	91,7 ± 2.1
	18,6	17,3 ± 0.3	1,8	93,2 ± 1.7
	46,5	44,9 ± 1.2	2,8	96,6 ± 3.0
	93,0	94,3 ± 3.6	3,8	101 ± 4.1
	223	227 ± 5.6	2,5	101 ± 2.5
	372	371 ± 5.4	1,4	99,8 ± 1.7
d18:1/18:1(9Z)	1,77	2,06 ± 0.1	6,0	116 ± 6.4
	4,43	4,47 ± 0.1	2,7	101 ± 2.8
	8,87	8,22 ± 0.3	3,5	92,6 ± 3.3
	17,7	16,5 ± 0.1	0,7	92,8 ± 0.6
	44,3	41,9 ± 1.5	3,5	94,6 ± 3.3
	88,7	91,3 ± 1.6	1,7	103 ± 1.7
	213	213 ± 1.0	0,5	100 ± 0.6
	355	355 ± 2.7	0,8	100 ± 0.8
d18:1/18:0	1,77	2,16 ± 0.1	5,0	122 ± 6.1
	4,42	4,64 ± 0.2	4,2	105 ± 4.0
	8,83	7,83 ± 0.2	2,7	87,8 ± 2.4
	17,7	15,4 ± 0.2	1,1	86,9 ± 1.0
	44,2	40,3 ± 0.7	1,8	91,2 ± 1.5
	88,3	92,0 ± 7.6	8,3	104 ± 8.9
	212	223 ± 10.7	4,8	105 ± 4.7
	353	347 ± 15.0	4,3	98,3 ± 4.6
d18:1/20:0	1,68	2,01 ± 0.1	3,8	119 ± 4.0
	4,21	4,24 ± 0.2	3,8	101 ± 3.5
	8,42	7,65 ± 0.1	0,9	90,9 ± 0.8
	16,8	15,0 ± 0.3	1,7	89,0 ± 1.5
	42,1	40,7 ± 2.1	5,2	96,4 ± 4.9
	84,2	87,1 ± 2.9	3,3	104 ± 3.6
	202	209 ± 7.7	3,7	103 ± 4.2
	337	332 ± 11.0	3,3	98,5 ± 3.4
d18:1/24:1(15Z)	1,54	1,75 ± 0.1	3,6	113 ± 3.8

	3,86	3,75 ± 0.1	5,5	97,4 ± 5.5
	7,71	7,04 ± 0.1	3,4	91,3 ± 3.2
	15,4	14,3 ± 0.5	4,5	92,5 ± 4.2
	38,6	39,0 ± 0.8	5,9	101 ± 6.0
	77,1	83,0 ± 2.9	3,5	108 ± 4.0
	185	189 ± 0.8	0,5	102 ± 1.0
	309	301 ± 3.9	1,4	97,5 ± 1.4
d18:1/24:0	1,54	1,89 ± 0.1	3,8	123 ± 4.6
	3,85	3,86 ± 0.1	3,2	100 ± 3.1
	7,69	6,87 ± 0.0	0,5	89,4 ± 0.5
	15,4	13,8 ± 0.1	1,1	89,8 ± 1.0
	38,5	36,5 ± 0.8	6,1	95,1 ± 6.0
	76,9	78,2 ± 3.8	4,8	102 ± 5.1
	185	191 ± 2.4	2,9	104 ± 3.1
	308	305 ± 5.7	2,6	99,1 ± 2.7

The indicated amounts of lipids were processed and analyzed at three consecutive days. Data are presented as mean ± SD. Accuracy is reported as the mean ± SD of the assayed concentration, in percent of nominal concentration., Coefficient of variation.CV, Coefficient of variation.
